# Predictors of Response to Intradetrusor Botulinum Toxin-A Injections in Patients with Idiopathic Overactive Bladder

**DOI:** 10.1155/2009/328364

**Published:** 2009-09-08

**Authors:** Brian L. Cohen, Daniel J. Caruso, Prashanth Kanagarajah, Angelo E. Gousse

**Affiliations:** Department of Urology, University of Miami Miller School of Medicine, Miami, FL 33101, USA

## Abstract

*Objectives*. To evaluate whether there are any demographic or urodynamic differences in patients with idiopathic overactive bladder (I-OAB) that respond and do not respond to intradetrusor injections of botulinum toxin-A (BTX-A). *Methods*. This represents a secondary analysis of data collected from an investigator initiated randomized trial designed to evaluate clinical differences in outcomes for 100 versus 150 U BTX-A in patients with I-OAB. Preinjection demographic and urodynamic data were collected. Patients were evaluated 12 weeks after injection and were determined to be responders or nonresponders as defined by our criteria. Statistical comparisons were made between groups. *Results*. In patients with overactive bladder without incontinence (OAB-Dry), there were no variables that could be used to predict response to BTX-A. On univariate analysis, younger patients with overactive bladder with incontinence (OAB-Wet) were more likely to respond to BTX-A than older patients. However, this relationship was no longer statistically significant on multivariate analysis. *Conclusions*. We were unable to identify any preinjection demographic or urodynamic parameters that could aid in predicting which patients will achieve clinical response to BTX-A. Future studies are necessary to further evaluate this question.

## 1. Introduction

I-OAB is a highly prevalent condition in both the United States and Europe with millions of adults being affected by the disorder [1]. Oral antimuscarinic agents have been the mainstay of treatment for patients with I-OAB; however they are associated with a high discontinuation rate either due to intolerable side effects or lack of efficacy. Alternative therapies in the form of sacral neuromodulation and more recently intradetrusor BTX-A injection are now becoming attractive alternatives for patients failing oral medications. 

As newer surgical treatment options become available, improved understanding of both the mechanism of action and the ideal patient for each treatment is paramount considering the cost involved. Currently, little is known concerning the ability to predict which patients will or will not respond to sacral neuromodulation [2], and even less is known about which patient will respond to BTX-A therapy [3–5]. Therefore, we conducted a secondary analysis of patients enrolled in a prospective randomized trial comparing 2 doses of intradetrusor BTX-A therapy for I-OAB in order to determine any demographic or urodynamic (UDS) parameters that may assist in predicting which patients will respond to BTX-A.

## 2. Materials and Methods

This study is a secondary analysis from an ongoing, investigator initiated, IRB-approved, randomized, prospective study to evaluate symptomatic and UDS improvement in patients with I-OAB receiving either 100 or 150 U of BTX-A (Botox, Allergan, Irvine, Calif, USA).

From January 2002 until January 2008, 47 patients with I-OAB were randomized to receive either 100 or 150 U of BTX-A at a single center. All patients signed an informed consent prior to study enrollment. The study protocol has been approved by our Institutional Review Board.

Patients with I-OAB as defined by the International Continence Society (ICS) were eligible for the study [6], and UDS demonstration of detrusor overactivity (DO) was not a requirement for study eligibility. Patients were subclassified into 2 groups: OAB-Wet and OAB-dry. As per a three-day voiding diary (3-VD), patients with urgency and frequency of urination (>8 voids per day) with at least one daily episode of urge urinary incontinence (UUI) were included within the OAB-wet group. OAB-Dry patients had urinary urgency, frequency >8 voids per day, and no complaint of UUI as documented by 3-VD. Subjects were required to have either failed or have been unable to tolerate treatment with at least 2 different antimuscarinic agents and have been treated with these agents for at least 2 months prior to enrollment. Subjects were required to discontinue all medications that could interfere with urethrovesical function 4 weeks prior to the first BTX-A injection.

Patients with a history of renal impairment, myasthenia gravis, neurogenic bladder dysfunction, bladder or kidney tumor, serious medical comorbidity, and pregnant or breast-feeding women were excluded from the study. Additionally, patients with bladder outlet obstruction as defined by a pressure-flow urodynamic study, patients with a postvoid residual (PVR) >100 mL, and patients with demonstrable stress urinary incontinence either by exam or on UDS were excluded from the study. Obstructive voiding on UDS was determined by the ICS nomogram for men only. All men with obstructive voiding were excluded. For women, we used the Blaivas-Groutz nomogram and classified obstructive voiding as a maximal detrusor pressure at maximum flow (pDetQMax) of ≥20 cm H_2_O and a maximal free flow rate (Qmax) of <12 mL/s [7].

All study subjects underwent a complete history and physical examination, multichannel video urodynamics, and completed a 3-VD prior to study enrollment. Urinary frequency (UF), UUI episodes, and volume voided were collected from the 3-VD. Baseline laboratory evaluation included a basic metabolic panel, urinalysis, and urine culture. When documented, urinary tract infections were treated and sterile urine cultures were documented prior to study enrollment. Urinalysis, urine culture, PVR, 3-VD data, and adverse events were obtained at clinic visits at 2, 6, 12, and 24 weeks postinjection.

Our injection technique is well tolerated and has been previously reported [8]. Briefly, subjects were injected in an office setting after the bladder was instilled with 40 mL of 1% lidocaine solution using a 14 French urethral Foley catheter. After 15 minutes, an Olympus 14 French flexible cystoscope was introduced into the bladder. The bladder was then distended with 100–200 mL of 0.9% normal saline. Using a 27-guage flexible Olympus injection needle (1050 mm working length with a 4 mm needle length), the supratrigonal detrusor muscle was injected with BTX-A 10 units/mL in 10–15 separate sites using 1 mL for each injection site. A prophylactic antibiotic was prescribed for 3 days postinjection. All subjects voided prior to discharge.

Primary outcome analysis focused on data from the 3-VD. UF is the dominant symptom in the OAB-Dry group and was calculated from the average number of daily voids recorded from the 3-VD. We considered a 40% reduction in UF to be a positive clinical response. UUI is the dominant symptom in the OAB-Wet group and was calculated from the average number of daily incontinence episodes recorded on the 3-VD. We considered a 50% reduction in UUI episodes to be a positive clinical response. Preinjection demographic and UDS parameters were compared within groups using a Student's *t*-test for continuous variables and Chi square analysis for discontinuous variables with a significance level of *α* < 0.05.

## 3. Results and Discussion

Of the 20 patients in the OAB-Dry group injected with BTX-A, 15 were responders and 5 were considered nonresponders. Of the 27 patients in the OAB-Wet group, 17 were considered responders and 10 were considered nonresponders. Amongst OAB-Wet patients, there was a statistically significant difference in age between the responders and nonresponders. There were no statistically significant differences noted between responders and nonresponders in regards to dose of BTX-A received, patient sex, nor any of the measured UDS parameters (Table 1).

On univariate analysis, patients with both OAB-dry and OAB-wet who responded to the therapy were found to be younger than those that did not achieve clinical response (55 ± 15 years versus 68 ± 13 years, *P* = .03). No UDS parameters were useful for prediction of clinical response although responders tended to have higher maximal detrusor pressure (49 ± 24 versus 29 ± 16 cm H_2_O, *P* = .06) (Table 1). When these variables were placed in a logistic regression model for multivariate analysis, neither were significantly associated with response to BTX-A injection (*P* = .14 for pDetMax and *P* = .13 for age).

As little as 10 years ago, patients with I-OAB had few options for treatment with only 2 available medications and no surgical options. As both the general public and practitioners become more aware of OAB and the potential impact on quality of life, more and more patients are seeking care for this condition. For patients that fail medical therapy, sacral neuromodulation and increasingly BTX-A therapy are becoming viable options. With more options available for treatment of OAB than ever before, an improved understanding of an individual's likelihood of responding to a given therapy will not only improve patient care but also decrease the expense of failing other costly therapies. With this in mind, we have performed this secondary analysis of our data from an ongoing clinical trial comparing 2 doses of BTX-A therapy in patients with I-OAB in order to improve the understanding of who is more likely to respond to intradetrusor BTX-A.

When analyzing our 2 subgroups in this study cohort, we were unable to identify any demographic or urodynamic parameters that predicted response in the OAB-Dry patients, and we found that younger patients with incontinence (OAB-Wet) were more likely to respond. Although it did not achieve statistical significance, those with higher pDetMax tended to respond better than those with lower pressures (*P* = .06). However, in a multivariate model, no significant predictors for response could be identified.

Few other investigators have performed similar evaluations of their data. In their report of the first 100 patients with I-OAB injected with 100 U BTX-A, Schmid et al. found that poor compliance, maximal cystometric capacity <100 mL, and bladder wall fibrosis on biopsy portended poor response to BTX-A [3]. In a study of patients with neurogenic OAB receiving 300 U BTX-A, no differences were found between the responders and nonresponders concerning clinical and urodynamic data [5]. Those that responded to the therapy tended to have a lesser degree of bladder wall fibrosis, but this did not achieve statistical significance. Finally, Sahai et al. evaluated 33 patients with idiopathic OAB receiving 200 U of BTX-A and identified 5 patients who had a poor response [4]. These investigators found that poor responders had significantly higher maximal detrusor pressures during detrusor overactivity, and all other investigated parameters were similar. However, the information in this study may be confounded by the fact that 45% of the patients continued to take anticholinergic medication at the time of injection, and 30% continued their medication 6 months after the injection. We believe further studies with a larger cohort of patients are required to precisely predict the responders and nonresponders for BTX-A therapy.

## 4. Conclusions

BTX-A therapy is becoming increasingly popular for the management of I-OAB in patients who have failed medical therapy. As more options become available for the treatment of this condition, being able to predict which therapy will provide an individual with the best chance of response will be clinically and economically beneficial. The present study suggests that demographic and urodynamic data are not helpful for determining who will and will not respond to BTX-A therapy. Additional investigation into this matter is important and will add valuable information concerning the treatment of I-OAB with BTX-A.

In addition, prospective randomized study powered to detect predictors of response to Botox will have significant clinical applications. Our study represents one of the first ever to investigate predictors of a successful response to BTX-A patients in idiopathic OAB.

## Figures and Tables

**Table 1:** Comparison of responders and nonresponders in patients with OAB-Dry and OAB-Wet: demographic and urodynamic data.

	OAB-DRY			OAB-WET		
Parameter (units)	Responders	Nonresponders	*P*-value	Responders	Nonresponders	*P*-value
*N*	15	5	NA	17	10	NA
PVR (mL)	24 ± 36	29 ± 36	.76	24 ± 25	24 ± 33	.93
MCC (mL)	242 ± 112	263 ± 124	.66	248 ± 147	227 ± 100	.71
pDetMax (cm H_2_O)	40 ± 17	34 ± 18	.47	49 ± 24	29 ± 16	.06
pVesMax (cm H_2_O)	62 ± 27	51 ± 12	.38	74 ± 32	60 ± 30	.24
Voided vol (mL)	279 ± 148	226 ± 174	.51	223 ± 155	171 ± 97	.24
pDetQMax (cm H_2_O)	24 ± 12	43 ± 48	.58	35 ± 20	23 ± 20	.23
QMax (mL/sec)	14 ± 6	13 ± 11	.91	15 ± 11	14 ± 7	.99
DO+	5/15	1/5	.58	12/17	8/10	.99
100 Units	6	4	.15	8	4	.77
150 Units	9	1		11	4	
Age	48 ± 15	59 ± 13	.11	55 ± 15	68 ± 13	.03
Number of males	5	0	.20	1	0	.89

PVR: postvoid residual; MCC: maximum cystometric capacity; pDetMax: detrusor maximum pressure; pVesMax: vesical maximum pressure; vol: volume; Qmax: maximum flow rate; DO+: presence of detrusor overactivity; mL: milliliters; cm H_2_O: centimeters of water; mL/sec: milliliters per second.

## Abbreviations

BTX-A: Botulinum toxin-A
I-OAB: Idiopathic overactive bladder
ICS: International Continence Society
OAB-Dry: Overactive bladder without incontinence
OAB-Wet: Overactive bladder with incontinence
3-VD: 3 day voiding diary
UUI: Urge urinary incontinence
PVR: Postvoid residual
UF: Urinary frequency
UDS: Urodynamics
MCC: Maximal cystometric capacity
pDetMax: Maximal detrusor pressure
pVesMax: Maximal vesical pressure
pDetQMax: Detrusor pressure at maximal flow
QMax: Maximal flow rate
DO: Detrusor overactivity.
